# Severe Warm Autoimmune Hemolytic Anemia With Anti-PO Antibody Positivity: A Diagnostic Dilemma in a Resource-Limited Setting

**DOI:** 10.7759/cureus.88444

**Published:** 2025-07-21

**Authors:** Suhas Kataveni, Sai Pranay Gottimukkala, Venkat Tej Sai Bodla

**Affiliations:** 1 General Internal Medicine, Gandhi Medical College and Hospital, Secunderabad, IND; 2 General Medicine, Srikara Hospital, Hyderabad, IND

**Keywords:** anti-ku antibodies, anti-po antibodies, anti-u1snrnp, immune-mediated hemolysis, overlap syndrome mixed connective tissue disease, systemic lupus erythematosus (sle), warm autoimmune hemolytic anemia

## Abstract

This is a diagnostically challenging case of a 45-year-old woman with severe anemia (Hemoglobin: 2.9 g/dL) and laboratory evidence of hemolysis. She was found to have positive anti-PO, anti-Ku, and anti-U1snRNP antibodies, raising suspicion for an autoimmune etiology such as systemic lupus erythematosus (SLE). However, this case did not fulfill the European League Against Rheumatism/American College of Rheumatology (EULAR/ACR) 2019 or Systemic Lupus International Collaborating Clinics (SLICC) 2012 classification criteria due to the absence of antinuclear antibodies (ANA) by indirect immunofluorescence (IIF) and incomplete immunological testing. The patient responded to packed red blood cell (PRBC) transfusions, corticosteroids, and a single dose of rituximab. This case highlights diagnostic complexity in autoimmune presentations, especially in resource-constrained settings, and emphasizes caution in interpreting rare autoantibodies.

## Introduction

Autoimmune hemolytic anemia (AIHA) is characterized by the production of antibodies against red blood cells (RBCs). It is a very rare condition, with an incidence of fewer than 1-3 cases reported per 100,000 per year [1]. AIHA cases are classified according to the temperature at which the antibodies show their characteristic reactions. Warm antibodies show stronger reactions at 37 °C and lose their affinity as the temperature decreases. Cold antibodies show stronger affinity at temperatures of 0-4 °C, and the affinity decreases as the temperature rises. Patients with AIHA generally present with low hemoglobin (Hb), elevated reticulocyte count, low haptoglobin, high lactate dehydrogenase (LDH), high indirect bilirubin, and a positive direct antiglobulin test (DAT). AIHA is often secondary to underlying conditions. While lymphoproliferative disorders account for half of warm and cold AIHA cases, autoimmune conditions are the next leading cause of warm AIHA [1].

Systemic lupus erythematosus (SLE) is a chronic autoimmune inflammatory syndrome with varied presentations and a variety of antinuclear antibodies (ANA) detected in the serum of patients [2]. SLE is often a secondary cause of warm AIHA. Anti-PO antibody is an anti-ribosomal P antibody, which was originally described by Elkon et al. in 1985 by Western blot [2]. Although anti-ribosomal P antibodies are highly specific to SLE, their sensitivity is relatively low - they are present in only about 15-40% of SLE patients, depending on ethnicity and detection method. This means that a large proportion of SLE patients will test negative, making it unreliable as a general screening tool [2]. This case report discusses a woman who had been suffering from anemia for six months but did not undergo detailed investigations due to lack of affordability and had her condition managed at a local hospital with multiple blood transfusions, iron, and folic acid supplements. Despite conservative management, her Hb levels continued to drop, and she presented to the hospital with an Hb of 2.9 g/dL for detailed investigations, which later revealed an ANA profile positive for anti-PO, anti-Ku, and anti-U1snRNP antibodies. Although SLE may present with AIHA, it remains a diagnosis of exclusion, and classification criteria are meant for research rather than strict clinical diagnosis. Anti-PO antibodies, while described as specific in some studies, are neither sensitive nor included in the current European League Against Rheumatism/American College of Rheumatology (EULAR/ACR) or Systemic Lupus International Collaborating Clinics (SLICC) criteria for SLE classification [1,2].

## Case presentation

A 45-year-old woman presented to the emergency department with fatigue, jaundice, black stools, and persistent anemia unresponsive to oral iron or transfusions for six months. Examination revealed pallor, icterus, oral ulcers, alopecia, and diffuse musculoskeletal pain, but no joint swelling or synovitis.

Initial laboratory tests (Table 1) showed hemoglobin (Hb) 2.9 g/dL, indirect hyperbilirubinemia (2.3 mg/dL), lactate dehydrogenase (LDH) 1200 U/L, and a positive DAT for immunoglobulin G (IgG). Peripheral smear revealed macrocytic anemia without schistocytes. Reticulocyte count was 6.8% (absolute count ~180,000/µL). The reticulocyte production index (RPI) was approximately 2.0, suggesting a compensatory bone marrow response. Serum haptoglobin was undetectable (<10 mg/dL). Testing for vitamin B12 and folate was not performed due to financial constraints, although macrocytosis and oral ulcers raised clinical suspicion of possible deficiency. The absence of these results limits the ability to definitively rule out nutritional anemia, which can mimic or coexist with autoimmune hemolysis and potentially confound the interpretation of the underlying etiology.

**Table 1:** Laboratory values

Parameter	Day 1	Day 2	Day 3	Day 4	Day 5	Day 6	Day 7	Normal range values in women
Hemoglobin	2.9 g/dL	7.8 g/dL	7.4 g/dL	7.8 g/dL	7.8 g/dL	7.6 g/dL	8.1 g/dL	12.0–16.0 g/dL
RBC count	0.64 mil/cmm	2.33 mil/cmm	2.2 mil/cmm	2.34 mil/cmm	2.28 mil/cmm	2.27 mil/cmm	2.48 mil/cmm	4.0–5.1 mil/cmm
MCV	127 fL	102 fL	101.4 fL	101.5 fL	103.7 fL	102.2 fL	102.4 fL	80–100 fL
Platelet count	4.1 lakhs/cumm	2.04 lakhs/cumm	1.5 lakhs/cumm	1.5 lakhs/cumm	1.3 lakhs/cumm	1.00 lakhs/cumm	1.15 lakhs/cumm	1.5–4.5 lakhs/cumm
Neutrophils	76%	78%	78%	85%	85%	84%	83%	40–70%
Lymphocytes	19%	15%	17%	15%	10%	09%	14%	20–40%
Direct Bilirubin	4.2 mg/dL	3.7 mg/dL	3.3 mg/dL	2.4 mg/dL	1.8 mg/dL	2.1 mg/dL	1.6 mg/dL	0–0.3 mg/dL
Indirect Bilirubin	2.3 mg/dL	3.9 mg/dL	3.5 mg/dL	2.8 mg/dL	2.6 mg/dL	1.4 mg/dL	0.8 mg/dL	0.2–0.8 mg/dL
Uric acid	9mg/dL	9.9 mg/dL						2.4–6.0 mg/dL
LDH	1200 U/L	1109 U/L	969 U/L	900 U/L	779 U/L	662 U/L	506 U/L	140–280 U/L
IgA	0.5g/dL	-	-	-	-	-	-	0.7–4.0 g/dL
IgG	1658mg/dL	-	-	-	-	-	-	700–1600 mg/dL
IgM	313mg/dL	-	-	-	-	-	-	40–230 mg/dL

**Figure 1:**
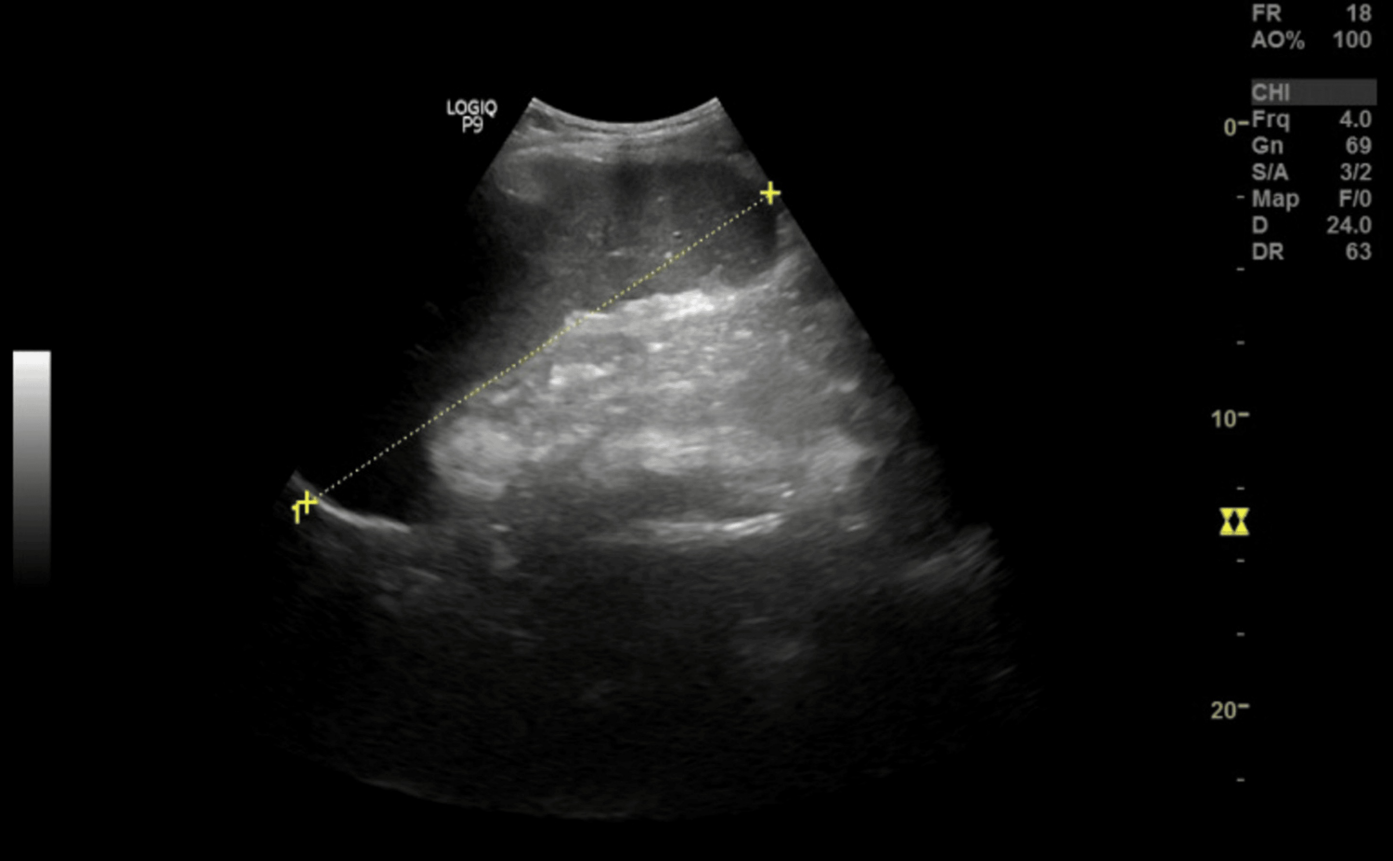
Enlarged spleen measuring 19 cms: moderate splenomegaly

**Figure 2:**
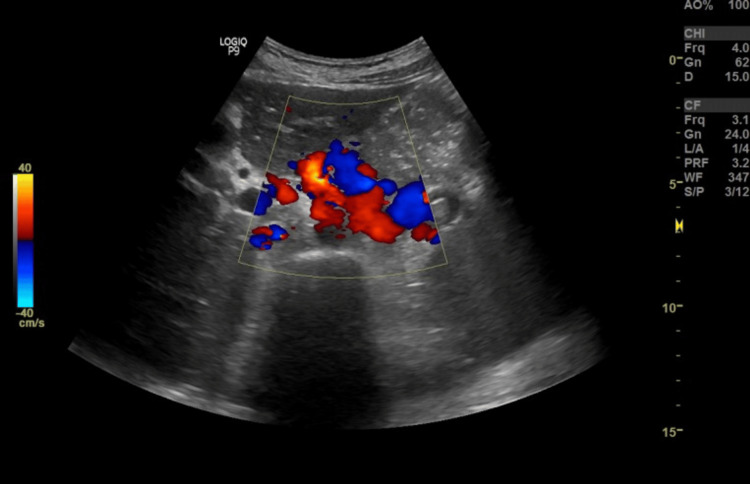
Multiple dilated and tortuous veins in the periportal region, suggestive of periportal varices

**Figure 3:**
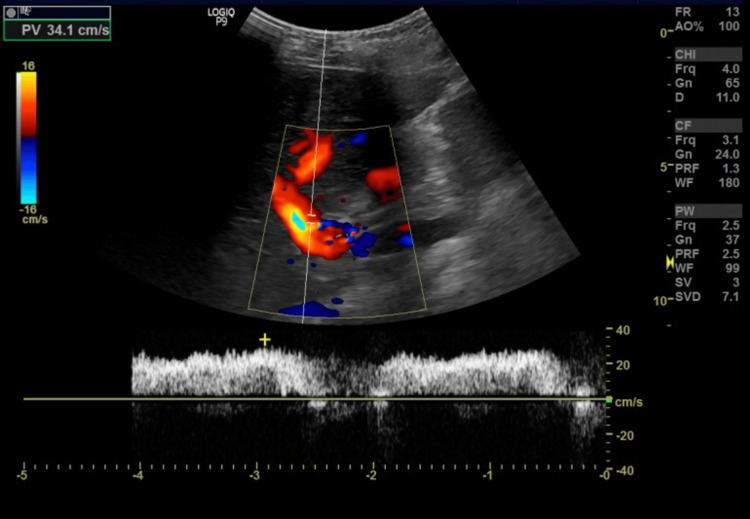
Portal vein Doppler showing normal hepatopetal flow with a normal peak systolic velocity of 34cm/sec. The portal vein diameter measured 13 mm (normal).

Abdominal ultrasound showed splenomegaly (19 cm) and periportal varices (Figures 1, 2). Despite this, Doppler studies demonstrated normal hepatopetal flow and a portal vein diameter of 13 mm with a peak systolic velocity of 34 cm/sec (Figure 3), suggesting non-cirrhotic portal hypertension or isolated splenic venous congestion.

Antinuclear antibody (ANA) testing by profile assay was negative for conventional markers (double-stranded DNA (dsDNA), Smith (Sm), Sjögren’s syndrome A (SSA), and Sjögren’s syndrome B (SSB)) but revealed strong anti-ribosomal P (anti-PO) and weak anti-Ku and anti-U1 small nuclear ribonucleoprotein (anti-U1snRNP) positivity. ANA by indirect immunofluorescence (IIF), serum complement levels, antiphospholipid antibodies, vitamin B12, folate, and soluble transferrin receptor (sTfR) were not tested due to financial limitations.

Differential diagnosis

The patient’s clinical and laboratory findings were indicative of hemolytic anemia, with hallmark features such as low hemoglobin (Hb), elevated indirect bilirubin, increased lactate dehydrogenase (LDH), a positive direct antiglobulin test (DAT), and splenomegaly. Warm autoimmune hemolytic anemia (wAIHA) was the most consistent diagnosis. However, other causes were considered. Evans syndrome was a possibility due to fluctuating platelet counts, although thrombocytopenia was not consistently present or below diagnostic thresholds. However, the coexistence of AIHA with even intermittent thrombocytopenia warranted consideration of this diagnosis, given its variable presentation and relapsing-remitting nature. Microangiopathic hemolytic anemia (MAHA) could not be completely ruled out but was considered unlikely given the absence of schistocytes on a manually reviewed peripheral smear and no overt thrombocytopenia. Paroxysmal nocturnal hemoglobinuria (PNH) was unlikely due to the presence of a positive DAT and lack of hemoglobinuria. Drug-induced hemolysis was improbable, as the patient reported no recent use of relevant medications. The late onset, lack of family history, and absence of characteristic smear features reduced the likelihood of hereditary hemolytic anemias such as hereditary spherocytosis or thalassemia. Hematologic malignancies, such as chronic lymphocytic leukemia (CLL) or myelodysplastic syndrome (MDS), could not be excluded definitively due to the absence of a bone marrow examination or flow cytometry. Lastly, while anti-Ku and anti-U1 small nuclear ribonucleoprotein (anti-U1snRNP) antibodies raised suspicion for mixed connective tissue disease (MCTD) or overlap syndromes, clinical features were insufficient to support these diagnoses at the time.

Treatment

The patient received four units of packed red blood cells (PRBCs), intravenous methylprednisolone 500 mg daily for three days, followed by oral prednisolone 30 mg twice daily. Due to the severity of anemia and modest steroid response, a single dose of rituximab (500 mg) was administered, based on the patient’s body surface area. Although the standard rituximab dosing is 375 mg/m² weekly for four weeks, a single-dose approach has been used in resource-constrained settings. Hydroxychloroquine 200 mg/day was initiated empirically.

Outcome

Hemoglobin stabilized between 7.6-8.1 g/dL during the first week. At follow-up, the patient reported improved well-being with hemoglobin rising to 10 g/dL. Platelet counts remained above 100,000/µL. Steroids were tapered over two weeks. Hydroxychloroquine was continued. No signs of recurrence were noted within the observation period.

## Discussion

This case highlights diagnostic uncertainty in autoimmune hemolysis, especially when access to tests is limited. A reticulocyte count and serum haptoglobin would have better confirmed hemolysis. SLE was suspected due to oral ulcers, musculoskeletal pain, hemolytic anemia, and rare autoantibodies; however, definitive classification criteria were not met due to negative ANA results [2]. Anti-PO antibodies, while specific, are not part of the SLICC or EULAR/ACR criteria and are insufficient for diagnosis. Similarly, anti-Ku and anti-U1 small nuclear ribonucleoprotein (anti-U1snRNP) antibodies are associated with mixed connective tissue disease (MCTD) or overlap syndromes, which were not fully evaluated here [2]. Rare autoantibodies, such as anti-PO, anti-Ku, and anti-U1snRNP, should not be overinterpreted in the absence of supporting clinical criteria, as they may occasionally be detected in other autoimmune conditions, transient inflammatory states, or even in asymptomatic individuals, underscoring the importance of clinical context in interpretation [2].

The possibility of hematologic malignancy was not excluded due to the absence of bone marrow examination or flow cytometry. MAHA was considered unlikely because manual peripheral smear review showed no schistocytes. Evans syndrome was considered but not diagnosed as the platelet count never fell below the diagnostic threshold. Iron supplementation was started empirically, but iron studies, including soluble transferrin receptor (sTfR), were not available to rule out iron overload. Ferritin was not used due to potential elevation from inflammation [3]. According to current international management guidelines for wAIHA, symptoms of anaemia should be addressed supportively through RBC transfusions, irrespective of the cause [4]. These transfusions help patients with wAIHA maintain their hemoglobin levels within clinically acceptable ranges until they can adjust to the reduced lifespan of RBCs, or until hemolysis subsides with treatment. The relative safety and efficacy of RBC transfusions may vary based on the severity of anaemia, class or functional activity of autoantibodies, associated symptoms, and any underlying conditions or comorbidities [4]. In this instance, the patient was administered 4 units of PRBC due to a critically low hemoglobin level of 2.9g/dl. Following the transfusion, the hemoglobin level increased to 7.8g/dl.

Systemic corticosteroids at a dosage of 1 mg/kg are the primary treatment for AIHA [5,6]. A randomized study strongly suggested that in first-line treatment, rituximab combined with steroids is superior to monotherapy with corticosteroids [6]. Treatment with rituximab was standard (single 500 mg dose) according to the patient’s body surface area, though the patient responded. Although the standard rituximab dosing is 375 mg/m² weekly for four weeks [7], a single-dose approach has been employed in resource-limited settings, with studies reporting favorable outcomes in autoimmune cytopenias, thereby supporting its clinical appropriateness [8]. The corticosteroids were initially administered intravenously as IV methylprednisolone (500 mg) and then transitioned to oral administration once an initial improvement was observed. The patient was also prescribed one dose of rituximab (500 mg) on day 3 of starting corticosteroid treatment. She showed clinical improvement after receiving transfusions and initiating pharmacotherapy. The initial dosage was continued for four weeks, during which the patient's hemoglobin levels stabilized between 7 and 8 mg/dl.

Hydroxychloroquine and chloroquine are antimalarial drugs licensed for the treatment of SLE [5]. The patient was discharged on tablet hydroxychloroquine 200 mg and tablet prednisolone 30 mg. In this scenario, initial oversight and conservative management resulted in misdiagnosis and deterioration of the condition, leading to a significant decrease in hemoglobin levels. A thorough evaluation of anemia with a comprehensive investigation is essential to identify the secondary causes of the disease. The presence of periportal varices with normal portal flow suggested splenic vein congestion rather than true portal hypertension. The reticulocyte production index (RPI) supported a functional marrow response. Vitamin B12 and folate deficiency remained a plausible but unconfirmed differential diagnosis. The patient’s improvement with immunosuppression supported a diagnosis of immune-mediated hemolysis.

## Conclusions

Severe anemia with hemolytic features requires prompt and comprehensive evaluation, including reticulocyte count and serum haptoglobin. Rare autoantibodies (anti-PO, anti-Ku, and anti-U1snRNP) should not be overinterpreted for diagnosis without supporting clinical criteria. Resource limitations may delay diagnosis and management in autoimmune conditions. Atypical autoimmune presentations require ongoing monitoring and periodic reassessment as more information becomes available.
